# Development of a Modified QuEChERS Method Coupled with LC-MS/MS for Determination of Spinetoram Residue in Soybean (*Glycine max*) and Cotton (*Gossypium hirsutum*)

**DOI:** 10.3390/jox13010002

**Published:** 2022-12-21

**Authors:** Arnab Goon, Chiranjit Kundu, Pritam Ganguly

**Affiliations:** 1 Department of Chemistry, University of Kalyani, Nadia 741235, India; 2 Department of Agricultural Chemicals, Bidhan Chandra Krishi Viswavidyalaya, Mohanpur 741246, India; 3 Department of Soil Science & Agricultural Chemistry, Bihar Agricultural University, Bhagalpur 813210, India

**Keywords:** spinetoram, soybean, cotton, dissipation, LC-MS/MS, PHI

## Abstract

An analytical method for the quantitative determination of the insecticide spinetoram in cotton and soybean was established and validated using liquid chromatography tandem mass spectrometry (LC-MS/MS). Spinetoram is the mixture of two spinosyns, 3′-O-ethyl-5,6-dihydro spinosyn J and 3′-O-ethyl spinosyn L. The method involves extraction with ethyl acetate followed by dispersive solid phase extraction (dSPE) clean-up with primary secondary amine (PSA), C_18_ and graphitised carbon black (GCB). The final quantitation of spinetoram was done by using LC-MS/MS with positive electrospray ionization. The method was reproducible (Horwitz ratio (HorRat) < 0.5 at 25 ng g^−1^) and validated by the analysis of samples spiked at 25, 50 and 100 ng g^−1^ in soybean, cotton and soil. The recoveries of spinosyns were found to be more than 85% when spiked at different levels. The identities of spinosyns were confirmed by using the ion ratio. A field dissipation study was conducted in soybean and cotton to find out the environmental fate of spinetoram, and samples were analysed following the proposed analytical method. Both isomers were found to be dissipated quickly. The pre-harvest interval of spinetoram was calculated in different substrates.

## 1. Introduction

Soybean (*Glycine max*) and cotton (*Gossypium hirsutum*) are two important crops cultivated in India. Due to the prevalence of a tropical climate across the country, both of these crops are attacked by numerous pests and pathogens that cause drastic yield losses annually. Pesticides play an important role in protecting the crops from these obnoxious pests and increase the yield to a significant extent. The use of new-generation insecticides in important crops like rice, cotton, pulses, vegetables, etc. has been getting encouraged to tackle new challenges set by insect pests. The key advantage of these new chemical compounds lies in the differences in their unique mode of action that enables them to act strongly in the field when used rotationally.

**Figure 1:**
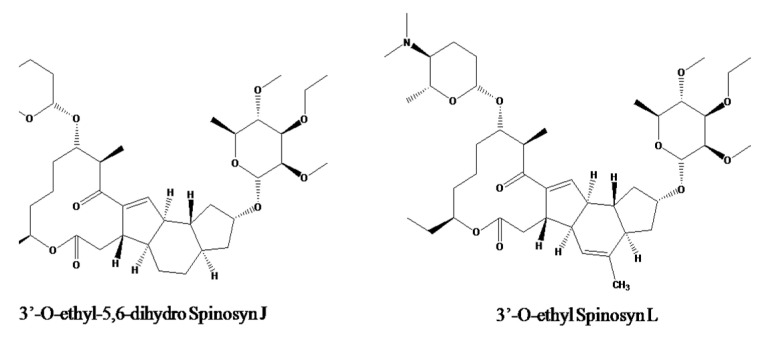
Chemical structures of 3′-O-ethyl-5,6-dihydro Spinosyn J (XDE-175-J) and 3′-O-ethyl Spinosyn L (XDE-175-L).

However, the major issue with respect to their use is the interaction of these “foreign compounds” with different components of the environment, especially the crop and soil matrix. These new compounds may act as a potential contaminant of soil and water bodies and may enter the food chain. There is every possibility of these new insecticides to create toxic effects in nontarget organisms, as in case of “spinetoram”. It was recently registered in India by Dow AgroScience Ltd. for use in crops like cotton, soybean and chili [1]. Spinetoram is a mixture (approx. 3:1 ratio) of two macrocyclic compounds 3′-O-ethyl-5,6-dihydro spinosyn J (spinosyn-J) and 3′-O-ethyl spinosyn L (spinosyn-L) [2] (Figure 1). Both spinosad and spinetoram are macrocyclic tetracycles connected to D-forosamine and rhamnose structurally [3]. The active ingredient spinosyns are derived from fermentation of a naturally occurring soil microorganism, *Saccharopolyspora spinosa*, followed by chemical modification to create the unique active ingredient spinosyn-J and spinosyn-L [4]. Spinosyns have a distinctive mechanism of action involving upset of nicotinic acetylcholine receptors [5,6,7,8]. Spinosyns have been found to be more selective, as compared to other existing insecticides, towards thrips, tobacco caterpillar and spotted boll worm that infest cotton [9] and soybean.

However, as mentioned earlier, the presence of pesticides in the soil can adversely affect soil health and its important chemical and biological functions. Furthermore, pesticides can enter the food chain by leaving toxic residues in the harvest and through contaminated drinking water as well, causing adverse impacts on nontarget organisms. Therefore, it is essential to monitor pesticide residues in food stuff and assess the risk to the consumer from a safety point of view.

The aim of this present study is to develop an analytical method, which is unavailable so far for these particular matrices, by which spinetoram, the mixture of spinosyn-J and spinosyn-L, can be determined at the trace level and to conduct field trials to study dissipation and residue at harvest of spinetoram in soybean and cotton for environmental and human safety. Liquid chromatography coupled with mass spectrometry has been widely used to determine compounds at a very low level [10,11] and is thus being employed in the study.

## 2. Materials and Methods

### 2.1. Apparatus

For the purpose of estimating the amount of spinetoram residue, an Alliance 2695 Separations Module (Waters, Milford, MA, USA) was used in conjunction with a Micromass Quattro Micro triple-quadrupole mass spectrometer (Micromass, Manchester, UK). MassLynx software V4.1 and QuanLynx were used to control the instruments and analyse the data, respectively. Using a Turbo Vap LV device from Caliper Life Science, the samples were evaporated (Hopkinton, MA, USA). A high-speed chilled centrifuge, Model Avanti J-30I, was used to centrifuge the extracts (Beckman coulter, Brea, CA, USA). The rotor heads could contain eighteen 10 mL (JA-21) and eight 50 mL (JA-30.50 T1) samples. The samples and powder reagents were weighed using a top-loading balance with a digital display (Sartorius, CP 225D, Göttingen, Germany). Fluorinated ethylene propylene (FEP) centrifuge tubes (Nalgene, Rochester, NY, USA) of 10 mL and 50 mL were used, respectively, for the extraction and the dSPE clean-up processes. The final extracts were stored in standard 1.8 mL dark glass autosampler vials. For sample preparation, a homogenizer (Polytron, PT-MR-3100, Kinemetica AG, Malters, Switzerland) as well as a pH metre (CL 46 type, Toshniwal Instruments Pvt. Ltd., Chennai, India) were also employed.

### 2.2. Reagents

From M/S Dow AgroSciences India Pvt. Ltd., Mumbai, certified reference material of spinetoram (99.2% pure) was purchased. Residue analysis-grade organic solvents from JT Baker (Phillipsburg, NJ, USA) were employed. These solvents included acetonitrile (ACN), methanol (MeOH), ethyl acetate (EA) and hexane. Using the Milli-Q (Millipore, Bedford, MA, USA) water purification system, purified water was obtained. Anhydrous sodium sulphate (Na_2_SO_4_), sodium chloride (NaCl) and ammonium acetate (CH_3_COONH_4_) were bought from Merck India Ltd. (Mumbai, India) for use as analytical reagents. Before usage, sodium sulphate was heated in a muffle furnace to 400–450 °C for five hours while being stored in a desiccator. Graphitized Carbon Black (GCB), Bondesil C_18_ and primary secondary amine (PSA; 40 µm particle size) were bought from United Chemical Technology (Bellefonte, PA, USA) and Varian (Harbor City, CA, USA), respectively. Bond Elute Amino (–NH_2_; Varian, Palo Alto, CA, USA) and florisil (60–100 mesh; Acros, Beel, Belgium) were also utilised in the analysis.

### 2.3. Preparation of Standard Solution

Stock solutions of spinetoram standard were made by weighing 10 mg in volumetric flasks (certified “A” class) and then dissolving it in 100 mL of methanol. Calibration concentrations of spinetoram containing spinosyn-J and spinosyn-L (5, 10, 20, 50, 100, 250, 500 and 1000 ng mL^−1^) were made following serial dilution of the stock solution using methanol. Three levels of fortification concentrations containing 25, 50 and 100 ng mL^−1^ were made by serial dilution of standard stock solution using methanol required for the recovery experiment.

### 2.4. Screening of Extracting Solvents and Adsorbents

Different organic solvents such as ACN, EA and EA–cyclohexane in the ratios of 9:1, 7:3 and 1:1 (*v*/*v*), respectively, were screened to find out the most efficient media for extraction of the analyte in the method. In case of adsorbents, the following combination options of different sorbents, PSA, Florisil, GCB, and C_18_ were compared to get improved analyte recovery and reduced matrix interference in soybean, cotton and soil—(i) no sorbent; (ii) 50 mg PSA; (iii) 50 mg PSA and 50 mg Florisil; (iv) 50 mg PSA and 50 mg C_18_; (v) 50 mg PSA, 20 mg GCB and 50 mg C_18_. In addition to the combination mentioned above, 300 mg of Na_2_SO_4_ was also added in every case. For the clean-up procedures, 4 mL of organic phase extract was used with the dispersive solid phase sorbent mentioned above.

### 2.5. Field Experiment

#### 2.5.1. Soybean

A field experiment was conducted with a randomised block design (RBD) to evaluate the persistence behaviour of spinetoram in soybean. Soybean plant was sprayed with spinetoram (12% SC; suspension concentrate) thrice based on the economic threshold level at a 10-day interval at 54 g a.i. ha^−1^ (T_1_) and 108 g a.i. ha^−1^ (T_2_) along with untreated control (T_3_), maintaining three replications per treatment. Soybean samples were collected randomly at 0 (2 h after application), 1, 3, 7 and 10 days after the final application (DAA). Field soil samples were collected randomly from a 0 to 15 cm layer at harvest. To avoid any breakdown of spinetoram residues before analysis, the samples were brought to the laboratory within dry ice bags where the samples were kept at −20 °C until analysis.

#### 2.5.2. Cotton

A similar field study was conducted in a cotton field with a randomised block design (RBD) to investigate the dissipation pattern of spinetoram in the crop. All the treatment doses, application timing and sampling interval remained same as mentioned in the case of the soybean field study (see Section 2.5.1).

### 2.6. Sample Extraction and Clean-Up Procedure

#### 2.6.1. Soybean, Cotton Plant and Soil

The following method was based on the original concept of the QuEChERS method reported by Anastassiades et. al., 2003 and modified accordingly [12]. The plant samples were finely chopped. Ten grams (10 g) of the chopped sample and soil were taken in a 50 mL centrifuge tube, and 10 mL of water was added, followed by a vortex for 1 min. Then, 10 mL of extracting solvent was added and then vortexed for 1 min followed by blending for 2 min at 10,000 rpm using a homogeniser. After that, 5 g of activated Na_2_SO_4_ and 1 g NaCl was added to the sample and again vortexed for 1 min. Following centrifugation of the sample at 5000 rpm for 10 min, 4 mL of the supernatant liquid was collected in a 10 mL centrifuge tube. Afterwards, a specific adsorbent combination and 300 mg of Na_2_SO_4_ were added to it and vortexed for 1 min, and the sample was again centrifuged for 10 min at 10,000 rpm. For soil samples, GCB was not used. Then, 2 mL of the liquid supernatant was taken from it and dried in a nitrogen evaporator at 40 °C. The residue was then restored in 2 mL of [MeOH: H_2_O (9:1, *v*/*v*) + 5 mM CH_3_COONH_4_]. The sample was then filtered through a 0.2 µm polyvinylidene fluoride membrane filter, and the filtrate was analysed in LC-MS/MS.

#### 2.6.2. Soybean and Cotton Oil

Grinded soybean and cotton seed (50 g) was extracted in a Soxhlet using 250 mL of hexane for six hours. The hexane fraction containing the oil was partitioned thrice with (100 + 50 + 50 mL) of acetonitrile, and the acetonitrile fraction was recovered over anhydrous Na_2_SO_4_. The combined acetonitrile phase was concentrated to 10 mL in a rotary vacuum evaporator at 40 °C. For clean-up, 4 mL of acetonitrile fraction was kept in a 10 mL centrifuge tube and followed the procedure described in Section 2.6.1. In this case, only GCB was not used.

#### 2.6.3. Soybean and Cotton De-Oil Cake

After extraction of oil, ten grams (10 g) of the de-oil cake was taken in a 50 mL centrifuge tube, and sample preparation done following the method described above (see Section 2.6.1) without GCB.

#### 2.6.4. Cotton Lint

A cotton lint sample (10 g) was Soxhleted using 120 mL of ethyl acetate for six hours. The ethyl acetate phase was evaporated in a rotary vacuum evaporator at 40 °C. The residue was then restored in 10 mL of [MeOH: H_2_O (9:1, *v*/*v*) + 5 mM CH_3_COONH_4_], filtered and analysed in LC-MS/MS.

### 2.7. LC-MS/MS Analysis

Residue detection and quantification were done by liquid chromatography-tandem mass spectrometry. The HPLC analysis was carried out by injecting 20 µL on a Symmetry C_18_ (5 µm; 2.1 × 100 mm) column (Waters, Milford, MA, USA) via an autosampler at a flow rate of 0.2 mL min^−1^. Retention times (RTs), qualifier ions, quantifier ions and ion ratios of both the isomers are depicted in Table 1. The mobile phase was constituted by mixing (A) methanol/water 10/90 (*v*/*v*) with 5 mM ammonium acetate and (B) methanol/water 90/10 (*v*/*v*) with 5 mM ammonium acetate. For analysis of both isomers, the samples were analysed using the mixture of 5% solvent A and 95% solvent B for 16 min. Direct infusion of spinetoram in methanol at a dose of 1 mg L^−1^ was used to select and tune transitions as well as analyte-dependent parameters. The optimised MS instrument parameters consisted of: cone voltage, 44 V; capillary voltage, 1.20 kV; source temperature, 120 °C; desolvation temperature, 350 °C; argon collision gas pressure to 3.5 e^−3^ psi; desolvation gas flow, 650 L h^−1^ nitrogen; cone gas flow, 25 L h^−1^ for MS/MS. By using three mass transitions for each test analyte and a dwell time of 0.150 s, multiple reaction monitoring (MRM) was used to estimate the residues.

### 2.8. Preparation of Matrix-Matched Calibration Standards

Eight concentration levels of spinosyn-J and spinosyn-L (5, 10, 20, 50, 100, 250, 500 and 1000 ng mL^−1^) were produced for calibration in LC-MS/MS. The supernatant from the blank sample after clean-up using the aforementioned method was utilised as the matrix solvent for calibration standards that matched the matrix. Matrix-matched standards were made by adding the proper amounts of the pesticide standard mixture to the blank extract in order to validate fortification tests.

### 2.9. Method Validation

The following validation parameters were taken into account when evaluating the method’s performance as per SANTE 11312/2021 guidelines [13].

Plotting the peak area versus the concentration of the relevant calibration standards at various calibration levels ranging between 5 and 1000 ng mL^−1^ provided the spinosyn-J and spinosyn-L calibration curves in pure solvent and matrix.

The limits of quantification (LOQs) were calculated by taking into account a signal-to-noise ratio of 10, while the limits of detection (LODs) were set by taking into consideration a signal-to-noise ratio of 3.

#### 2.9.1. Precision

The precision in terms of repeatability (six samples were processed by two different analysts each on a single day) and intermediate precision (six samples on six different days were processed by two different analysts) were worked out separately for a spiked concentration of 25 ng g^−1^ in soybean, cotton and soil. For both isomers, the Horwitz ratio (HorRat), which measures intralaboratory precision and reveals if a method is acceptable in terms of precision [14,15], was computed as follows:

HorRat = RSD/PRSD

where RSD stands for relative standard deviation, PRSD stands for predicted RSD = 2C^−0.15^, and C denotes the concentration indicated as a mass fraction (25 ng g^−1^ = 25 × 10^−9^).

#### 2.9.2. Recovery Experiments

The recovery experiments were conducted on fresh untreated cotton, soybean and soil by spiking the samples in six replicates with spinetoram at three concentration levels, i.e., 25, 50 and 100 ng g^−1^. The mixtures were extracted, cleaned up and analysed using the method mentioned above.

#### 2.9.3. Matrix Effect

The matrix effect (ME) was evaluated by using matrix-matched standards. Comparisons were made between the slopes of the calibration graphs based on the pure-solvent-based standards and the matrix-matched standards of soybean, cotton and soil. Signal amplification caused by the matrix was shown by a larger slope of the matrix calibration, whereas signal suppression was indicated by a lower slope. The following equation was used to evaluate the matrix effect (ME, %):

ME = [(peak area of matrix-matched standard − peak area of solvent standard) × 100]/peak area of solvent standard

According to the aforementioned equation, the ME’s negative and positive values represent, respectively, matrix-induced suppression and augmentation.

### 2.10. Pre-Harvest Interval (PHI)

PHI can be defined as the time gap between the final spray of a particular insecticide and the crop harvest. It is determined on the basis of the maximum residue limit (MRL) values of the insecticide in that particular crop in order to facilitate international trade [16,17]. PHI can be calculated with the help of the following formula.

PHI = [Ln (initial deposit) − Ln (MRL)]/slope of the regression equation

### 2.11. Calculation of Uncertainty

Three different sources of uncertainty were calculated, such as Type A uncertainty due to repeatability, Type B uncertainty due to individual components and thereby combined uncertainty and expanded uncertainty. The expanded uncertainty (U_exp_ at 95% confidence level) was determined at the 25 ng g^−1^ level for each matrix by using a coverage factor of 2 as per the method described in different literature [18,19,20]. Eight different sources of uncertainty were considered, i.e., (i) uncertainty due to repeatability using six replicates (U_a_), (ii) weighing balance (U_b1_), (iii) measuring cylinder of 100 mL for preparation of a stock solution (U_b2_), (iv) a micropipette of 1 mL for serial dilution (U_b3_), (v) certified reference material (U_b4_), (vi) a volumetric flask of 10 mL for preparation of intermediate stocks (U_b5_), (vii) a centrifuge at 5000 (U_b6_) and 10,000 (U_b7_) rpm and (viii) a calibration curve (U_b8_).

## 3. Results

### 3.1. Selection of the Extracting Solvent

**Figure 2:**
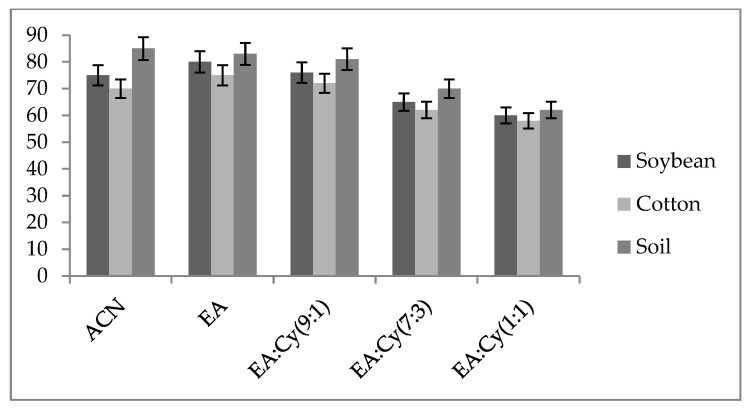
Comparison of extraction efficiency of different solvent systems spiked at 25 ng g^−1^ for spinosyn-J.

ACN, EA and EA–cyclohexane at ratios of 9:1, 7:3 and 1:1 (*v*/*v*) were evaluated for their extraction efficiency. In the case of EA, the recoveries of these macrocyclic compounds, spinosyn-J and spinosyn-L were higher than 85% when determined using matrix-matched standards. With ACN extraction, the outcome was found above 75% but not better than that with EA. Meanwhile, with EA–cyclohexane (9:1, *v*/*v*), the recoveries of the spinosyns were less than 85%. The recovery percentage did not significantly increase—instead, it decreased—as the cyclohexane proportion in the extracting solvent mixture (EA–cyclohexane; 7 + 3 and 1 + 1, *v*/*v*) was increased. Therefore, it was found that extraction with EA resulted in better recovery with improved precision (Figure 2). Precision in terms of HorRat at the 25 ng g^−1^ level was found below 0.5 for spinosyns in all the test matrices (Table 2), indicating satisfactory performance of the methodology. A literature survey reveals ethyl acetate (EA) as an efficient extracting solvent beside acetonitrile for different matrices [19,21,22,23].

### 3.2. Comparison of Shaking versus Blending versus Vortexing

To determine the ideal preliminary extraction process to be used for soybean, cotton and soil samples, the extractability of spinosyn-J and spinosyn-L residues was evaluated by comparison of shaking, blending and vortexing. It was evident that blending gave better recovery for spinosyns compared to that with vortexing- and shaking-based methods. For the soil matrices, the homogeniser was not used because higher recovery was found using the vortex method. Thus, blending using a homogeniser for extraction of spinosyn residues was adopted for soybean and cotton. For soil, the adoption of a vortex for the initial extraction was preferred.

### 3.3. Comparison of Different SPE Sorbents by LC–MS/MS Analysis

**Figure 3:**
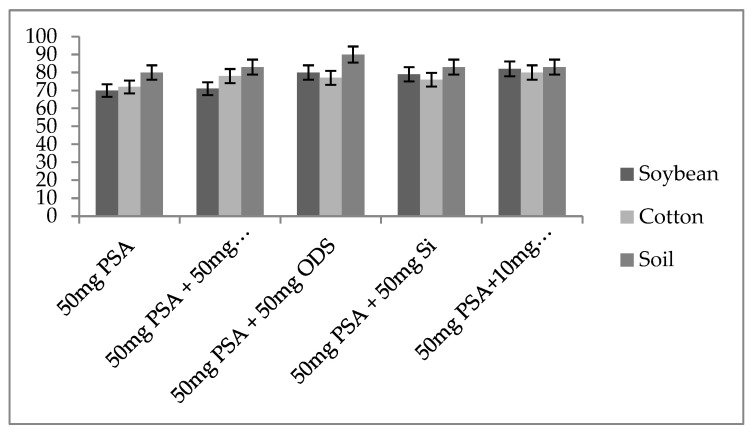
Clean-up capabilities of different d-SPE sorbents for spinosyn-J at 25 ng g^−1^ spiking level.

The primary goal of the clean-up step was to use various sorbents to eliminate those co-extractives from the extract as much as feasible. Weak ion exchange is one of the sorbents that is most frequently utilised (PSA or –NH_2_), Florisil, GCB and/or C_18_ SPE sorbent [24,25,26,27]. The recoveries achieved using combination no. (v) of 50 mg PSA, 50 mg C_18_ and 20 mg GCB gave the better result (Figure 3). Therefore, the combination of PSA, C_18_ and 20 mg GCB worked well as a clean-up sorbent for eradication of different co-extractives from the matrix.

Both spinosyn-J and spinosyn-L could be analysed by a single chromatographic run of 16 min. Spinetoram could be detected at 10 ng g^−1^ with the instrumental conditions used in this experiment. Linearity of the calibration curve was established for both spinosyns. The correlation coefficient (R^2^) derived from the calibration curve, both pure-solvent-based as well as matrix-matched were ≥0.99 for both isomers. The LOQs for spinosyn-J and spinosyn-L were 7.5 ng g^−1^ and 2.5 ng g^−1^, respectively. The matrix-induced suppression in target signals was present for the compound, which could have occurred due to suppressions in the ionization process. For soybean, cotton and soil matrices, the slopes of the two equations derived from matrix-matched calibration and pure-solvent-based calibrations were dissimilar. The matrix effect was notable for the macromolecule (spinosyn-J and spinosyn-L) compounds. To prevent any over- or underestimation of residues, the matrix-matched calibrations were employed for the relevant matrix-based quantification reasons, taking into account the matrix effects for spinosyns. Given that both spinosyns were examined in six replicates, the relative standard deviation for the methodology was less than 15%, which was fairly acceptable. High clean-up effectiveness and low matrix influence could be achieved with d-SPE clean-up by PSA + C_18_ + GCB, allowing application of this targeted and sensitive technique for routine analysis of spinetoram with acceptable recovery (80–110%).

**Figure 4:**
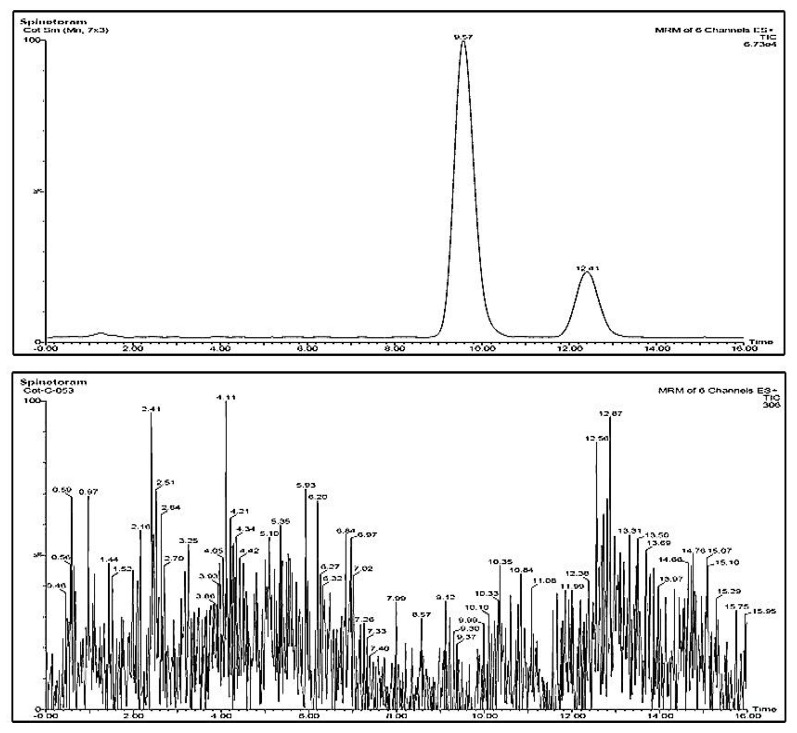
LC-MS/MS chromatogram of spinetoram recovered from cotton when spiked at 25 ng g^−1^ (**above**) and chromatogram of control cotton sample (**below**).

**Figure 5:**
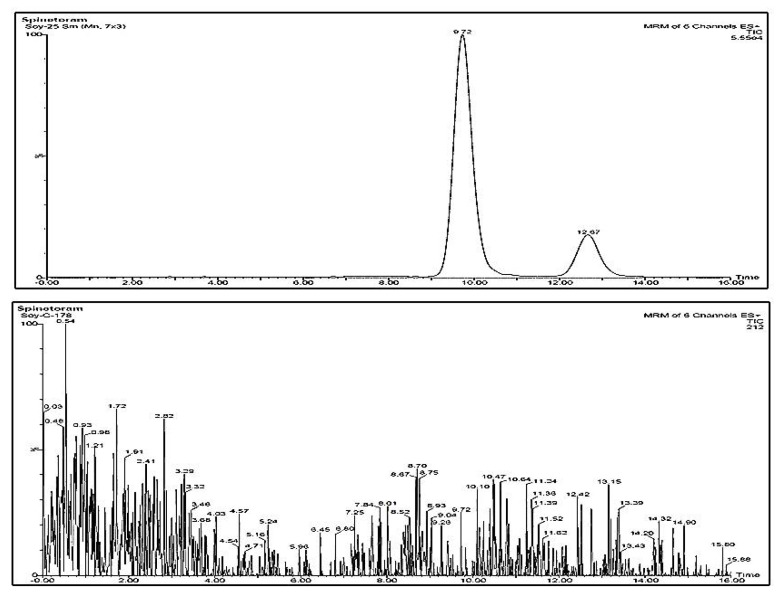
LC-MS/MS chromatogram of spinetoram recovered from soybean when spiked at 25 ng g^−1^ (**above**) and chromatogram of control soybean sample (**below**).

No interfering compound peaks were noticed at the specific retention time of spinosyns in chromatograms derived from control blank cotton (Figure 4), soybean (Figure 5) and soil samples.

### 3.4. Result of Field Study

The field samples were analysed using the abovementioned method. The identity of spinetoram in field samples was confirmed by the qualifier-to-quantifier ratio within a 30% tolerance range of the corresponding matrix-matched standard.

The findings of the field study of persistence of spinetoram in soybean are summarised in Table 3. It was observed that the residues declined progressively with time following first-order kinetics, irrespective of doses. The initial deposits (2 h after spraying) of spinosyn-J present in spinetoram were 0.249 μg g^−1^ (T_1_) and 0.572 μg g^−1^ (T_2_) in soybean, and the half-life values (T_1/2_) were 1.58 d (T_1_) and 2.09 d (T_2_). In the case of spinosyn-L, the initial deposits (2 h after spraying) were 0.114 μg g^−1^ (T_1_) and 0.194 μg g^−1^ (T_2_), and the half-life values (T_1/2_) were 1.17 d (T_1_) and 1.85 d (T_2_). No residue of spinetoram was detected in harvest samples of soybean oil, de-oil cake and soil.

The field study results for cotton showed a decreasing trend of residue with time as with soybean, following first-order kinetics (Table 3). The initial deposits of spinosyn-J present in cotton were 0.232 μg g^−1^ (T_1_) and 0.504 μg g^−1^ (T_2_), and the half-life (T_1/2_) values were 1.42 d (T_1_) and 1.91 d (T_2_). The initial deposits of spinosyn-L were 0.104 μg g^−1^ (T_1_) and 0.165 μg g^−1^ (T_2_). The half-life (T_1/2_) values of spinosyn-L were 1.07 d (T_1_) and 1.47 d (T_2_). In the harvest samples of cotton, which includes cotton oil, de-oil cake, cotton lint and soil, no residue of spinetoram was detected.

### 3.5. PHI of Spinetoram

MRL values of spinetoram (spinosyn J and spinosyn L) in soybean, soybean oil and cottonseed oil have been fixed by FSSAI, Govt. of India as 0.02 parts per million (mg kg^−1^ or mg L^−1^) [28]. PHI values in soybean were found to be 6.04 d and 9.91 d for T_1_ and T_2_, respectively. In the cases of soybean oil and cottonseed oil, spinetoram residues were found to be below the detectable limit of cumulative LOQ, i.e., 0.010 mg kg^−1^, which was much lower than the prescribed MRL value.

### 3.6. Estimation of Uncertainty

Expanded uncertainty was calculated on the basis of various sources of uncertainty and is presented in Table 2. It was found that estimated U_exp_ values for both isomers in different matrices were below 10%. It shows that the method adopted for sample processing and analysis was efficient enough and suitable for determination of spinetoram in both crop matrices and soil [20].

## 4. Discussion

Pesticide degradation is strongly influenced by biotic (micro- and macroflora) and abiotic (environmental) variables (soil, water, temperature, solar energy, etc.). Wide variations in the degradation pattern of the chemicals may result from any modifications to these parameters [29,30,31,32]. In an experiment dealing with six different pesticides, including spinetoram in pak choi, it was reported that dissipation of the compounds was faster in open field conditions than in greenhouse conditions due to several environmental factors, including rainfall and sunlight. A PHI value of 6 d has been determined for spinetoram in pak choi crop in the study [33]. According to the US EPA, spinetoram residues dissipate very rapidly under aquatic field conditions with a reported half value ≤ 1. It is also being reported that spinosyn J in particular is less persistent in the environment due to degradation caused by both biotic and abiotic factors. This particular isomer is stable to hydrolysis at environmental pH (5–9) but undergoes photolysis rapidly, resulting in shorter half-life [34,35]. Biodegradation is also considered as the major driving factor under aerobic conditions for quick dissipation of spinetoram. Both isomers under terrestrial field conditions have very shorter half-life values [34]. In perennial crops such as tea, spinetoram has been found to be less persistent in fresh tea leaves, resulting in shorter half-life values of 0.70 d. The transfer rates of the compound from fresh tea leaves to processed tea and consequently in infusion range between 34.9% and 57.8% and 36.9% and 68.2%, respectively [36]. In another study, half-life values of spinetoram were found to be 2.82 d in soil, 5.77 d in cabbage stem, 4.21 d in roots and 3.57 d in cabbage leaf when applied as seed-palletised coating [37]. An analytical method based on acetonitrile extraction and PSA as adsorbent was found suitable for analyzing spinetoram residues in red bayberry and soil. The calculated half-life values were found to be in the range of 4.4–5.2 and 1.2–1.9 d in the crop and soil, respectively. The recorded amount of terminal residues was lower than the prescribed MRL value (1 mg kg^−1^) of spinetoram in red bayberry [38]. Similar observations were found in various studies where spinetoram half-life values were reported as 1.29 d in cabbage, 1.95 d in pepper [39], 2.17 d in pear fruits [40], 2.6 d in tomato [41], 4.85 d in cauliflower [42] and 1.1 d in rice straw [16]. Faster dissipation of spinetoram from the plant surface may also be attributed to volatilization of the insecticide from the crop surface [41]. The observation was supported by another study where half-life values of spinetoram were found to be 2.4–3.0 d and 2.8–4.0 d in crown daisy and sweet pepper, respectively [43]. A safe waiting period of 11 d has been recommended before harvesting of tomato after application of spinetoram 12% SC at 240 mL ha^−1^ in Egypt [44], and the corresponding value is 7 d for sweet cherry in the United States [45].

## 5. Conclusions

A cheap, easy, robust and effective method was developed to analyse spinetoram residues in soybean, cotton and soil which was not being done so far. The method can detect spinetoram residues at the trace level, which is below the MRL values of the compound in the substrates. Spinetoram can dissipate quickly in soybean and cotton under the mentioned experimental conditions. A safe waiting period, i.e., a PHI of 6 d for the recommended dose of spinetoram in soybean following Good Agricultural Practices (GAP) should be maintained.

## Figures and Tables

**Table 1:** Overview of LC–MS/MS monitoring of the spinosyns.

Pesticide	RT (min)	Q	Q_1_	CV (V)	CE (V)	Q_2_	CV (V)	CE (V)	Ion Ratio (%) (Mean ± RSD)
Spinosyn-J	9.53	748.87	142.1	44	29	97.9	44	60	17 ± 8
Spinosyn-L	12.28	760.83	142.1	44	28	95.7	44	65	1 ± 9

**Table 2:** Recovery % (RSD) ^a^, expanded uncertainty (%) at 25 ng g^−1^, HorRat and matrix effect of test pesticides.

Substrate	Level of Fortification (ng g^−1^)									
	Spinosyn-J					Spinosyn-L				
	25	50	100	HorRat ^b^	ME (%) ^c^	25	50	100	HorRat ^b^	ME (%) ^c^
Soybean plant	86 (11) [7.86]	89 (10)	88 (7)	0.41	−59	85 (10) [7.45]	87 (9)	87 (6)	0.35	−48
Soybean oil	81 (11) [6.19]	85 (8)	88 (10)	0.40	−37	82 (9) [5.35]	86 (7)	90 (6)	0.33	−34
De-oil cake	86 (12) [6.03]	90 (7)	87 (9)	0.43	−44	85 (12) [6.54]	91 (8)	87 (7)	0.42	−38
Cotton plant	86 (11) [7.02]	90 (9)	89 (8)	0.40	−49	85 (11) [6.11]	89 (7)	90 (6)	0.40	−40
Cotton oil	83 (7) [6.86]	86 (8)	88 (6)	0.25	−51	90 (10) [6.54]	86 (8)	92 (8)	0.34	−46
De-oil cake	87 (8) [5.98]	85 (7)	92 (6)	0.28	−32	85 (8) [7.31]	88 (9)	93 (6)	0.28	−41
Cotton lint	89 (9) [7.37]	95 (10)	92 (7)	0.33	−11	89 (7) [7.41]	95 (9)	91 (5)	0.26	−18
Soil	89 (12) [6.08]	93 (7)	94 (6)	0.42	−16	88 (7) [3.22]	91 (4)	91 (6)	0.25	−26

^a^*n* = 6. ^b^ HorRat measured at 25 ng/g. ^c^ ME (%) shows matrix-induced signal suppression (“−”sign) or enhancement.

**Table 3:** Dissipation of spinetoram residue in soybean and cotton.

Days after Application	Treatment	Residues of Spinetoram (mg kg^−1^) (Mean ± SD) (Dissipation %)			
		Soybean		Cotton	
		Spinosyn J	Spinosyn L	Spinosyn J	Spinosyn L
0	T_1_	0.249 ± 0.007 (-)	0.114 ± 0.010 (-)	0.232 ± 0.007 (-)	0.104 ± 0.007 (-)
	T_2_	0.572 ± 0.008 (-)	0.194 ± 0.007 (-)	0.504 ± 0.007 (-)	0.165 ± 0.005 (-)
1	T_1_	0.186 ± 0.008 (25.3%)	0.066 ± 0.007 (42.10%)	0.153 ± 0.006 (34.05%)	0.048 ± 0.003 (53.84%)
	T_2_	0.435 ± 0.010 (23.95%)	0.130 ± 0.007 (32.98%)	0.404 ± 0.006 (19.84%)	0.104 ± 0.006 (36.96%)
3	T_1_	0.068 ± 0.007 (72.69%)	0.019 ± 0.003 (83.33%)	0.055 ± 0.004 (76.29%)	0.015 ± 0.002 (85.57%)
	T_2_	0.212 ± 0.008 (62.93%)	0.073 ± 0.007 (62.37%)	0.194 ± 0.007 (61.50%)	0.042 ± 0.003 (74.54%)
7	T_1_	BDL	BDL	BDL	BDL
	T_2_	0.050 ± 0.008 (91.26%)	0.009 ± 0.002 (95.36%)	0.042 ± 0.003 (91.66%)	0.006 ± 0.001 (96.36%)
10	T_1_	BDL	BDL	BDL	BDL
	T_2_	BDL	BDL	BDL	BDL
**Regression equation (R^2^)**	T_1_	Y = 5.575 − 0.440X (0.986)	Y = 4.753 − 0.593X (0.999)	Y = 5.474 − 0.486X (0.997)	Y = 4.595 − 0.645X (0.995)
	T_2_	Y = 6.391 − 0.352X (0.999)	Y = 5.355 − 0.433X (0.987)	Y = 6.302 − 0.362X (0.996)	Y = 5.044 − 0.452X (0.997)
**Half-life (T_1/2_) (d)**	T_1_	1.58	1.17	1.43	1.07
	T_2_	1.97	1.60	1.91	1.53

BDL = below detectable limit; <0.0075 µg g^−1^ (spinosyn-J), <0.0025 µg g^−1^ (spinosyn-L).

## Data Availability

Not applicable.
